# [^64/67^Cu]-FAP theranostics in combination with immunotherapy in a preclinical model of TNBC

**DOI:** 10.21203/rs.3.rs-10790344/v1

**Published:** 2026-09-08

**Authors:** Sharmila Sridhar, Volkan Tekin, Addison Hunt, Shannon E. Lynch, Carlos A. Gallegos, Julienne L. Carstens, Suzanne E. Lapi, Anna Sorace

**Affiliations:** University of Alabama at Birmingham; University of Alabama at Birmingham; University of Alabama at Birmingham; University of Alabama at Birmingham; University of Alabama at Birmingham; University of Alabama at Birmingham; University of Alabama at Birmingham; University of Alabama at Birmingham

**Keywords:** PET, SPECT, FAP, autoradiography, targeted radionuclide therapy, breast cancer

## Abstract

**Rationale:**

Fibroblast activation protein (FAP) is a prominent transmembrane protein in the tumor microenvironment (TME) that promotes tumorigenesis. FAP has been a recent target for radionuclide-conjugated peptides for PET imaging and targeted radionuclide therapy (TRT). The objective of this study is to develop a theranostic approach using [^64/67^Cu]-FAP2287 in combination with immunotherapy (IMT) to treat triple-negative breast cancer (TNBC).

**Methods:**

4T1 tumor-bearing BALB/c mice (N = 20) were injected and imaged with 100μCi [^64^Cu]-FAP2287 PET at 1, 4, and 24h to quantify baseline FAP expression followed by autoradiography. 4T1-tumor bearing BALB/c mice (N = 42) were divided into Control, IMT, 800μCi [^67^Cu]-FAP2287 or 4)[^67^Cu]-FAP2287 + IMT and 1600μCi [^67^Cu]-FAP2287, or [^67^Cu]-FAP2287 + IMT (N = 5–10/group) and imaged with [^64^Cu]-FAP2287 PET followed by treatment. All [^67^Cu]-FAP2287 groups were imaged with SPECT at 4, 24, and 48h. Tumor growth and survival rates were monitored. Standard uptake values (SUV) quantified, statistical analysis and probability of survival were done with significance at p < 0.05.

**Results:**

At baseline, there was a strong positive correlation between [^64^Cu]-FAP2287 PET SUV_Mean_ and autoradiography (R^2^ = 0.7806,p < 0.001). No changes in [^67^Cu]-FAP2287 retention over time was observed following the IMT. [^64^Cu]-FAP2287 SUV_Mean_ proved to be a reliable predictor of survival with 800μCi (R^2^ = 0.84,p = 0.03) and 1600μCi (R^2^ = 0.62,p = 0.01). FAP-TRT increased survival compared to controls and IMT. 1600μCi [^67^Cu]-FAP2287 + IMT significantly decreased tumor volume (p = 0.0003) and increased survival (p = 0.0005) compared to [^67^Cu]-FAP2287.

**Conclusion:**

We can noninvasively target and predict response to FAP radiolabeled with elementally paired theranostic probe, [^64/67^Cu]-FAP2287 in TNBC. IMT synergistically increased response to FAP-TRT and can guide personalized approaches to improve treatment efficacy.

## INTRODUCTION

Triple-negative breast cancer (TNBC) represents about 15–20% of breast cancer and does not overexpress the hormone receptors estrogen (ER), progesterone (PR) and human epidermal growth factor (HER2) receptors and is one of the leading causes of cancer related deaths in females (1). 2.3 million breast cancer cases are reported every year and 15–20% of those reported cases are categorized as TNBC (2–4). With no overexpression of targetable cell membrane proteins, the standard-of-care for TNBC includes chemotherapy, radiation, and more recently, immunotherapy (IMT) is being incorporated at both early and late-stage disease (5–9). Despite inducing increases in T cell activation and infiltration (10–13), IMT only improves the prognosis in a portion of TNBC patients due to the ever-evolving tumor microenvironment (TME) (14). In particular, the TNBC TME is composed of several pro- and anti-tumorigenic factors which contribute to therapeutic response including stromal cells, blood vessels, tumor-associated macrophages, extracellular matrix (ECM), and cancer associated fibroblasts (CAFs) (15–18). CAFs play a pivotal role in cancer metastasis, ECM remodeling, therapeutic resistance, angiogenesis, decreased immunogenicity, and increased mesenchymal transition (19, 20). CAFs and tumor cells with high mesenchymal transition express fibroblast activation protein (FAP), a type II membrane-bound glycoprotein belonging to the dipeptidyl peptidase family (21). Studies have previously demonstrated that high FAP expression is correlated with increased local tumor invasion, increased risk of lymph node metastases, and decreased survival of patients with several types of cancer (22). As there is a clinical need to create new combination strategies for treatment, clinical and pre-clinical studies have explored targeting FAP for targeted radionuclide therapy (TRT) to treat several types of solid tumors that are hard to treat with standard-of-care (23).

TRT induces double-stranded DNA breaks causing immunogenic cell death (ICD) but can also lead to immunomodulation and increased infiltration of immune cells. Others have demonstrated that radiation, whether through external beam or TRT, can increase response to IMT across various tumor types (24–27). Theranostic pairs for FAP TRT have been primarily developed using radionuc0lides such as [^68^Ga, ^18^F] for diagnosis and [^177^Lu] for therapy. However, these radionuclides have inherently different half-lives, chemistry, and chelators which can significantly influence the pharmacological behavior of the radiotracer, resulting in different distribution profiles (28, 29). For personalized TRT strategies, pre-treatment dosimetry studies are performed with the diagnostic radionuclide of the theranostic pair to optimize injected activity and predict the therapeutic absorbed doses, distribution, accumulation, metabolism, elimination and decay rates in tissues of interest. It has been shown that both the chelator and the isotope alter the distribution when using the same targeting probe (30–32). Elementally paired isotopes, such as [^64^Cu] and [^67^Cu], have similar chemistry and pharmacokinetic profiles, which could address the limitations of the current gold standard [^68^Ga/^177^Lu] in theranostic applications. [^64^Cu], with a half-life of 12.7 h, can be used for diagnostic imaging via positron emission tomography (PET), while [^67^Cu], with a half-life of 2.58 days, decays by β - and γ-rays which can be used for therapy and imaged using single photon emission computed tomography (SPECT) imaging (33).

The objective of this study is to develop a theranostic approach to establish FAP as a targeting molecule for treating TNBC in syngeneic mouse models of disease that have intact immune systems, validate [^64/67^Cu] radionuclide pair targeting FAP in TNBC, and evaluate its synergistic effects with IMT. Evaluating the relationship of chemically-paired theranostic approaches targeting FAP and optimizing radiopharmaceutical development has an opportunity to improve patient care, increasing both therapeutic effectiveness and predictive capabilities on a personalized basis [30]. FAP2287 is a murine surrogate to FAP2286, a clinically relevant FAP peptide used in theranostic clinical trials (e.g. NCT04939610, NCT06880757, NCT04621435). FAP2287 is a cyclic peptide proven to have sensitivity to FAP with improved properties such as binding affinity and plasma stability to mouse (34, 35). We hypothesize that [^64^Cu] radiolabeled with FAP2287 will be predictive of treatment outcomes to [^67^Cu]-FAP2287 treatment and that the addition of IMT after FAP-TRT will increase therapeutic response. Our study represents some of the first work looking at Cu radiolabeling of FAP2287, chemically paired [^64/67^Cu] for FAP theranostics, and combining IMT with FAP-TRT for the treatment of TNBC. Overall, our study aims to investigate novel elementally matched theranostic agents, modulate the tumor microenvironment to optimize combination targeted treatments, and improve overall survival in TNBC preclinical models.

## METHODOLOGY

### Cell Culture and Tumor Model Development

4T1 (mouse TNBC cell line syngeneic to BLAB/c mice) cells were acquired from ATCC (catalog number: CRL-2539-LUC2) and cultured in Roswell Park Memorial Institute (RPMI) 1640 Medium (Fisher, 11-875-119) supplemented with 10% fetal bovine serum (FBS) and 1% penicillin-streptomycin. E0771 (mouse TNBC cell line syngeneic to C57BL/6 mice), TS/A (mouse ER+ cell line syngeneic to BALB/c mice) and TUBO (rat HER2+/neu cell line syngeneic to BALB/c mice) purchased from ATCC (Manassas, VA), syngeneic breast cells were cultured in Dulbecco’s Modified Eagle’s Media (DMEM) supplemented with 10% FBS, 1% Sodium Pyruvate and 1% L-Glutamine. All cells were maintained at 37^°^C and 5% CO_2_. Cells were maintained at passage numbers below 25 at a confluency of 75–80%. Cells were evaluated for FAP expression via western blot. Female 6- to 12-week-old BALB/c mice (Charles River Laboratories) were injected with 2x10^5^ 4T1 cells suspended in phosphate buffer saline in the right 3rd mammary fat pad for development of an orthotopic model of TNBC. Tumor volumes of 45-140mm^3^ (9 days post inoculation) were enrolled in the study. Mice (N = 42) were randomly divided into 6 treatment groups: namely, 1) Control (N = 6), 2) IMT (N = 6), 3) 800μCi [^67^Cu]-FAP2287 (N = 5), 4) 800μCi [^67^Cu]-FAP2287 + IMT (N = 6), 5) 1600μCi [^67^Cu]-FAP2287 (N = 9), or 6) 1600μCi [^67^Cu]-FAP2287 + IMT (N = 10). Animals were treated with one dose of 50μl [^67^Cu]-FAP2287 via intravenous (IV) retro-orbital injection, either 100μl IMT (100μg Anti-CTLA4 (InVivoMab BE0164) +200μg Anti-PD1(InVivoMab, BE0146)), or 100μl saline via intraperitoneal (IP) injections on day 0, 3, 6 and 9. Tumors were measured every other day with calipers and tumor volume was calculated using the formula $\left(\frac{4}{3}*3.14*\left(\frac{l}{2}\right)*\left(\frac{b}{2}\right)*\left(\frac{l+b}{4}\right)\right),$ to monitor longitudinal changes. Mice were categorized into responders and non-responders based on tumor volume changes using the immune response evaluation criteria in solid tumors (iRECIST) with a 20% decrease in tumor volume from baseline were considered responders. Endpoints for tumor-bearing animals include sustained tumor volume of 1500 mm^3^ or 20% loss of body weight.

### Radiochemistry of Copper-64 and Copper-67 labeling of FAP2287

Copper-64 [^64^Cu] was produced via the ^64^Ni(p,n)^64^Cu reaction. Nickel-64 (Isoflex, San Francisco, CA, USA) was electroplated into a 1mm thick gold backing and placed behind a 1mm aluminum degrader. The target was bombarded at 18MeV on the degrader (13MeV on target) and 40uA for 2–4 h. After the bombardment, the target was dissolved in 9M HCl, loaded on a 2.5g AG1x8 resin column, and washed with 15mL of 9M HCl. Co-produced cobalt-61 was eluted with 4mL of 4M HCl (36). ^64^Cu was eluted with 5mL of 0.1M HCl, dried at 100°C under vacuum, and reconstituted in 0.1M HCl. Copper-67 [^67^Cu] was purchased from The Idaho Accelerator Center, Idaho State University, Pocatello, ID.

4μg of FAP2287 (clovis Oncology) (in aqueous solution, 1mg/1mL) mixed with 50mM ammonium acetate (2:1, v/v) and 0.1M gentisic acid (1:1, v/v) and either 0.1mCi (37MBq) ^64^Cu or 0.8mCi (296MBq) of [^67^Cu] for 15min at 400 rpm at 95°C. The radiolabeling yield was assessed using thin layer chromatography (TLC) with 50mM DTPA solution and iTLC (glass microfiber chromatography paper impregnated with a silica gel, Agilent).

### Biological validation of FAP expression in 4T1 TNBC model

#### In Vitro Western Blot Assessment

A range of syngeneic breast cancer cells subtypes (TNBC, ER+, HER2+) were cultured (4T1, TUBO, E0771 and TS/A) and lysed for 10 min with radioimmunoprecipitation assay (RIPA) buffer (Roche Applied Science, Indianapolis, IN, USA) on ice to isolate the proteins. Protein concentrations from all samples were determined using the Bicinchoninic Acid (BCA) Protein assay. Samples were run and probed using Abcam rabbit FAP primary antibody (EPR20021) 1:1000 overnight and with anti-rabbit secondary IgG antibody with the dilution of 1:1000 for 3 h (37). The film was developed for 30 min, and the bands were visualized using Fiji ImageJ software (public domain JAVA image processing program, National Institute of Health, Bethesda, MA). FAP expression of each cell line was normalized to β-actin expression and analyzed.

#### In Vivo [^64^Cu]-FAP2287 PET for Imaging

4T1 FAP+ tumor bearing BALB/c mice (N = 20) were intravenously injected with 4μg/100μCi of [^64^Cu]-FAP2287 and imaged at three different time points (1, 4, and 24 h) post-injection with pre-clinical small animal PET/CT (Sofie Biosciences GENXT, Dulles, VA) to optimize FAP2287 nuclear imaging. Anesthesia was maintained with 2% isoflurane in air, and body temperature was maintained at 36°C. 20-min static PET was acquired followed by a 5-min CT for anatomical reference. DICOMs with Bq/ml were converted to standardized uptake values (SUV) using the formula $SUV=\frac{concetration\;of\;the\;radiotracer\;x\;body\;weight}{decay\;corrected\;dose}$ (VivoQuant Version 5.2, Invicro, Needham). Regions of interest (ROIs) including the tumor and contralateral quadricep muscle were manually identified with CT and [^64^Cu]-FAP2287 uptake were quantified for those regions using SUV_mean_, SUV_max_, SUV_median_, SUV_peak_, Tumor-to-muscle ratio (TMR) and histogram analysis.

#### Ex Vivo Autoradiography

Following [^64^Cu]-FAP2287 PET imaging, (N = 20) mice were euthanized, and tumors were excised and sliced into 1mm sections. All the slices were placed on a cassette (GE Healthcare) along with standards (1nCi, 2nCi and 10nCi). A film was placed on a cassette and exposed for 3 hours in the dark. The film was then placed in an Amersham Typhoon Scanner, and a Phosphor image was obtained (sensitivity, 4000; pixel size, 50μm). Autoradiography scans were then calibrated and quantified (VQ Autoradiography Calibration Tool). ROIs of the tumor sections were semi-automatically generated using global thresholding and regional smoothing. Mean, max, and peak autoradiography concentrations were calculated for the combination of individual tumor sections (38).

### Theranostic paired [Cu]-FAP2287 Nuclear Imaging

4T1 tumor bearing BALB/c mice were imaged with 100 μCi [^64^Cu]-FAP2287 4 h post-injection as previously described. The following day, mice were administered with 800μCi or 1600μCi [^67^Cu]-FAP2287 therapeutics and underwent SPECT images for 30 min followed by CT using (MILabs U-SPECT^6^, Netherlands) at 4, 24 and 48 h post-injection. A phantom scan with a known concentration at 0.8mm voxel with nine iterations and no post filtering (MILabs) was used to identify a calibration factor and SPECT images were reconstructed. The reconstructed images were then attenuation corrected with the CT. SPECT images were filtered through a gaussian filter of 0.6mm, decay corrected and converted from μCi/cc to SUV (VivoQuant). ROIs of tumor and muscle (background) were segmented with CT and [^67^Cu]-FAP2287 uptakes per region were quantified. Mice weights were regularly taken and monitored for any physical changes.

### Pilot investigation of metastasis following treatment with bioluminescence Imaging (BLI)

Mice with longstanding survival (> 75 days) following treatment protocols were injected with 100μl D-luciferin (115144-35-9, GoldBio, Olivette, MO) via intraperitoneal injection and a 5-min bioluminescence was acquired (IVIS Lumina III (Perkin Elmer, Waltham, MA). Quantification of total flux (p/s) was performed in primary tumor injection sites and visible areas with metastasis (Living Image, Waltham, MA). Mice were rechallenged with 2x10^5^ 4T1 tumors left 3rd mammary fat pad and the tumors were measured to assess tumor growth [38].

### Statistical Analysis

Two-tailed unpaired t-test and one-way ANOVA were used to determine the differences in [^64^Cu]-FAP2287 uptake between the two imaging groups to determine retention of radiotracer over time. Multiple t-tests with adjusted α values were used to evaluate the differences in tumor volume of different treatment groups over time. Kaplan Meier’s estimator was used to analyze the probability of survival. Pearson’s correlation was used to determine the correlation between SUV values and autoradiography values and correlation between the probability of survival and [^64^Cu]-FAP2287 SUV. P values < 0.05 were considered significant. The Bliss test of synergy was used to identify the synergistic effects of the combination therapy (39, 40). Mice were categorized into responders and non-responders based on tumor volume changes using the immune response evaluation criteria in solid tumors (iRECIST) (41).

## RESULTS

### In vitro, in vivo and ex vivo biological validation of FAP expression in 4T1 TNBC model

Baseline protein expression of FAP in different syngeneic mouse breast cancer cell lines, including TS/A, 4T1, E0771 and TUBO was quantified (Supplemental Fig. 1A), showing that 4T1 (TNBC) tumor cells have increased FAP expression compared to the other syngeneic breast cancer cell lines, (Supplemental Fig. 1B).

The uptake and retention of [^64^Cu]-FAP2287 in the tumor was evaluated using PET at 1, 4, and 24 h post-injection. Transverse images from the static scan (Fig. 1A), reveal that there is an uptake of radiotracer in the tumor, which is retained through 24 h. Qualitatively, there is heterogeneity in the spatial distribution of the radiotracer in the tumor both *in vivo* imaging and *ex vivo* autoradiography. *Ex vivo* autoradiography revealed increased retention in the peripheral regions compared to the core. Autoradiography showed a positive correlation to PET imaging (Fig. 1B), with SUV_mean_ values being positively correlated with the mean values from autoradiography (R^2^ =0.7806, p < 0.001).

### Tumor uptake of chemically paired radioisotopes [Cu] conjugated with FAP2287

To understand the correlation of pharmacokinetics between ^64^Cu and ^67^Cu, 4T1 tumor bearing BALB/c mice were imaged with [^64^Cu]-FAP2287 followed by [^67^Cu]-FAP2287 SPECT within 24 hrs, as shown in the coronal images of [^64^Cu]-FAP2287 PET and [^67^Cu]-FAP2287 SPECT (Fig. 2A). These images demonstrate the heterogeneity of both radiotracer uptake in the tumor, with specific regions having high [^64^Cu]-FAP2287 and [^67^Cu]-FAP2287 uptake and [^67^Cu]-FAP2287 retention over time. Using the average SUV_mean_ of the tumor across the cohort as a threshold, [^64^Cu]-FAP2287 uptake per tumor was stratified into two groups demonstrating high and low uptake (Fig. 2B). When comparing the high and low [^64^Cu]-FAP2287 groups to its respective [^67^Cu]-FAP2287 uptake, there was a significantly higher amount of [^67^Cu]-FAP2287 in the high PET signal cohort compared to the low cohort (p = 0.026) (Fig. 2C).

### [^67^Cu]-FAP2287 in combination with IMT demonstrates synergistic response

[^67^Cu]-FAP2287 SPECT images (Fig. 3A) across time points show an average of 51.33 ± 16.53% washout of the radiotracer from the tumor region by 48h. There was no significant differences [^67^Cu]-FAP2287 retention measured by SUV changes, in 48h following the administration of IMT compared to tumors that did not receive IMT (Fig. 3B–C). Mice were imaged and treated following the timeline displayed in (Fig. 4A). IMT alone demonstrated no differences in tumor curves compared to saline control, confirming the tumor is not responsive to checkpoint inhibitors alone (p > 0.05). A tumor growth curve shows therapeutic response from both the doses (800/1600 μCi) of [^67^Cu]-FAP2287 with and without the addition of IMT (Fig. 4B and D). Mice treated with 800 μCi [^67^Cu] + IMT had significantly reduced tumor burden compared to the controls (p = 0.002) by day 17 post treatment (Fig. 4B). Mice treated with 1600μCi [^67^Cu]-FAP2287 compared to controls had significantly reduced tumor volumes (p = 0.0004) and combination group had significantly reduced tumor volumes compared to the controls (p = 0.00025), IMT (p = 0.00066), and [^67^Cu]-FAP2287 (p = 0.0014) (Fig. 4D) by day 17 from the beginning of treatment. When comparing longitudinal survival on day 52, the 800μCi [^67^Cu]-FAP2287 group had a significantly increased probability of survival compared to the controls (p < 0.01). The combination group of 800μCi [^67^Cu]-FAP2287 plus IMT had significantly increased probability of survival compared to controls (p < 0.001) and IMT alone (p = 0.04) (Fig. 4C). The 1600μCi [^67^Cu]-FAP2287 group showed significantly higher probability of survival compared to control (p = 0.0053). The combination treatment cohort (1600μCi [67Cu]-FAP2287 plus IMT) had a significantly higher probability of survival compared to controls (p < 0.0001), IMT (p < 0.01) and [^67^Cu]-FAP2287 (p < 0.01) (Fig. 4E). The Bliss test of synergy done at 5 different time points (on days 1, 6, 9, 11 and 16 from treatment) revealed synergy in the combination treatment with both the higher and lower dose of [^67^Cu]-FAP2287 with the higher combination group tumor volume having significantly reduced tumor volumes compared to the Bliss test line (p = 0.006 Fig. 4F and G). Using the iRECIST criteria we observed that the response rate was 33.3% from 800μCi [^67^Cu]-FAP2287, 66.7% from 800μCi [^67^Cu]-FAP2287 + IMT, none from 1600μCi [^67^Cu]-FAP2287 and 70% from 1600μCi [^67^Cu]-FAP2287 + IMT treated groups. The response rate for controls and IMT treatments was 0%. We observed any changes in body weight or physiological changes in these treated mice, we observed no physical changes or decrease in body weight over time (supplemental Fig. 2).

### [^64^Cu]-FAP2287 PET imaging as a reliable predictor for response to [^67^Cu]-FAP2287 TRT

To analyze if [^64^Cu]-FAP2287 PET can be a reliable predictor of overall survival to [^67^Cu]-FAP2287, [^64^Cu]-FAP2287 SUV_mean_ were plotted against probability of survival of the mice that received 800/1600μCi [^67^Cu]-FAP2287 alone (not in combination with IMT) to observe if there is a correlation between the two entities. The SUV values were positively correlated with days of survival in both the lower and higher dose of [^67^Cu]-FAP2287 dose with 800μCi (Fig. 5A, R^2^ = 0.84, p = 0.03) and 1600μCi (Fig. 5B, R^2^ = 0.62, p = 0.01), suggesting [^64^Cu]-FAP2287 PET is a reliable predictor for long-term response to [^67^Cu]-FAP2287 TRT but there was a decrease in correlation between probability of survival and [^64^Cu]-FAP2287 SUV_mean_.

### Exploratory preliminary assessment of long-term anti-tumor response

To assess metastatic tumor burden (as 4T1 tumors metastasize to organs such as lungs, liver, brain, etc.) (42), we performed BLI imaging on long-standing survivors on day 75 from tumor implantation and observed that for our combination treatment, there were N = 1 mouse/group (out of remaining 7 from 1600 and 4 from 800μCi) with no primary tumors. On day 88 from tumor implantation, N = 1 per lower and higher doses had achieved a complete response with no recurrence or metastasis of the primary tumor, which was confirmed using BLI (supplemental Fig. 3A). These mice were rechallenged with tumor cells (exploratory), and tumor growth was monitored closely every two days. We observed that the mouse that had received that 1600μCi showed increased resistance to secondary tumor growth and significantly decreased rates of tumor growth compared to the mouse that received 800μCi (p < 0.0001) which had relapse in primary tumor growth (supplemental Fig. 3B).

## DISCUSSION

FAP has been defined as a good target for solid tumors across many cancer types, which propelled translational theranostic trials and success of those trials across many cancers including TNBC. FAP2286 is a cyclic peptide which is a human equivalent of FAP2287 and is already in several clinical trials, and cyclic peptides have demonstrated higher binding affinity, specificity, and retention in the tumor region compared to the linear peptides [40]. However, there have been limited pre-clinical studies demonstrating combination of TRT approaches with IMT. This work builds upon what others, such as Zboralski, D et. al. previously showed where [^177^Lu]-FAP2287 studied preclinically with transduced MCA205-mFAP cells and paired [^68^Ga] PET imaging revealed increased enhanced response to anti PD-1 following FAP TRT [41]. Our study builds upon this work with different dosing of TRT in a syngeneic model with naturally available overexpression of FAP and using novel chemically-similar theranostic pairs. Further, we demonstrate long-standing response using combination approaches of FAP-TRT and checkpoint inhibitors in tumors that are resistant to immunotherapy alone (35, 43).

Our studies build upon the growing work of others that have previously demonstrated robust responses of breast cancer to TRT in preclinical studies. TRT causes DNA damage that leads to immunomodulation of TME and induces ICD, which leads to increased infiltration of CD4 + and CD8 + T cells in the TME [44–46]. Brenda Gibbens-Bandala B, et. al, used [^177^Lu]-Bombesin-PLGA (paclitaxel) for treatment of TNBC human-cell line (MDA-MB-231) tumors and revealed synergy through increased higher cell death in combination with chemotherapy, paclitaxel. Kasten BB, et. al, showed the use of [^212^Pb]-225.28 for reduced tumor burden in the TNBC human cell line xenografts. Breast cancer is known to have an immunogenically cold TME with low infiltrating T cells. Due to its low immunogenicity, TNBC has varied response to IMT which have been shown both clinically and preclinically (44–46). With the addition of immunotherapy, there is an increase in activation and infiltration of T cells and other effector cell populations, which mediate cell death and enhance therapeutic effects. However, very few studies have evaluated the use of combination therapies with TRT and IMT. Few such studies reviewed the current standard [^177^Lu] TRT with IMT in several pre-clinical studies including Kleinendorst, S. C., et. al, and Kerr, C. P., et. al, compared several preclinical and clinical studies with β [^177^Lu], α [^212^Pb] TRT and external beam radiation in combination with IMT and observed an increase in anti-immune response with TRT with further ideas to optimize TRT dosing in combination with IMT (26, 47). Similar were observed in a preclinical study using immunotherapy response syngeneic tumors, E0771 in C57/B16 mouse model, treated with IMT in combination with ^177^Lu TRT showing increased survival and decreased tumor volumes in the combination group compared to the other treatment groups (48). Our work compliments these and expands upon their work, demonstrating [^67^Cu]-FAP2287 TRT in combination with IMT in a model that is not responsive to IMT and revealed reduced tumor volume, increased the probability of survival, and almost doubled the survival rate of mice with aggressive 4T1 TNBC tumors.

Eisazadeh, R., et. al, showed that high tumor-to-background ratio of [^68^Ga]Ga-PSMA-11 PET can be a significant prognostic for [^177^Lu]Lu-PSMA therapy and associated with improved survival in a clinical study. Ozkan, E., et. al, observed that [^68^Ga]Ga-PSMA PET is a good way to predict patients who will benefit from [^177^Lu]Lu-PSMA treatment based on pretreatment SUV metrics that were all statistically significant factor in prediction of PSA-based response and RECIP disease control in clinical settings (49, 50). Other clinical studies Sharma, R., et. al, and Lee, H., et. al, showed similar trends for predicting response to peptide receptor radionuclide therapy, [^177^Lu]-DOTATATE in neuroendocrine tumors using [^68^Ga]-DOTATATE PET (51, 52). Our study bridges the gap between the current treatment options, IMT, with new elementally-paired FAP TRT using [^64/67^Cu]-FAP2287 TRT to enhance the efficacy of IMT and to identify predictive approaches to personalize treatment in a preclinical syngeneic TNBC model. We successfully were able to demonstrate that [^64^Cu]-FAP2287 PET can predict response to [^67^Cu]-FAP2287 TRT alone and can be used as a diagnostic tool to stratify groups based on baseline FAP expression for increased response to TRT in preclinical model of TNBC. Cingoranelli, S, et. al, previous showed that with the use of elementally paired isotopes in pre-clinical models of prostate cancer [^43^Sc]Sc-PSMA-617 was able to predict probability of survival for both low and high doses of [^47^Sc]Sc-PSMA-617 treatment. We successfully were also able to demonstrate that [^64^Cu]-FAP2287 PET can predict response to [^67^Cu]-FAP2287 TRT alone and can be used as a diagnostic tool to stratify groups based on baseline FAP expression for increased response to TRT in pre-clinical model of TNBC (53). However, one drawback of this study design was that the survival data for the combination treatment mice was censored following the death of mice from other treatment groups. Therefore, we could not correlate the [^64^Cu]-FAP2287 PET values to the probability of survival for the combination group to assess if [^64^Cu]-FAP2287 PET can be a reliable predictor of response to [^67^Cu]-FAP2287 + IMT (supplemental Fig. 4A and B). Theranostic approaches using [^67^Cu]-FAP2287 in combination with IMT provide an opportunity to non-invasively target a critical component of the TME to enhance clinical decision making for treating TNBC. Lower and higher doses of [^67^Cu]-FAP2287 successfully reduced tumor burden and enhanced the efficacy of IMT in 4T1 tumor-bearing mice, which led to an increase in the probability of survival. With the higher dose of [^67^Cu]-FAP2287, there is a higher rate of ICD which enhances the therapeutic effects of IMT and successfully reduces the tumor burden compared to the lower dose of [^67^Cu]-FAP2287, with no visible side effects We observed a correlation between [^64^Cu]-FAP2287 SUV_mean_ to days of survival when treated with [^67^Cu]-FAP2287 TRT. We also observed that our combination therapy with both 800 and 1600μCi doses of [^67^Cu]-FAP2287 and IMT had synergistic effects resulting in enhanced treatment response and reduced tumor volumes with the higher dose of [^67^Cu]-FAP2287. These results emphasize the importance of combination therapy approaches to modulate the TME and overcome treatment resistance in TNBC. Importantly, there is very little work done in establishing a relation between the diagnostic and therapeutic agent for predicting response. We identified [^64^Cu]-FAP2287 as a good diagnostic tool to differentiate between responders and non-responders of [^67^Cu]-FAP2287 TRT to streamline the process of treating TNBC cases with TRT and IMT, allowing to stratify TRT based on baseline FAP expression for enhanced treatment efficacy.

Despite exciting preclinical results in preclinical response and improve therapeutic efficacy, future directions are still needed to optimize [^67^Cu]-FAP2287 dosing, possibly identifying the lowest amount of [^64/67^Cu]-FAP2287 that can be used and to identify how multiple doses of delivered TRT affect combination therapy. Identifying the best dosing strategy could further optimize patient care and identify opportunities for best tumor burden eradication (and reduction of future metastasis). This study only used one dose of TRT and could have potentially further increased effectiveness pr reduced metastatic burden by optimizing the dosing or personalizing dose amount based on PET imaging. Additionally, this study shows an exploratory investigation into long-term survival and re-challenging. There is a need to characterize the mechanism behind the potential long-term immune surveillance and the effect of memory T cells in stunting primary tumor growth following [^64/67^Cu] FAP2287 TRT. We observed that animals with complete disappearance of the primary lesion following combination treatment had decreased tumor growth when rechallenged. Studies exploring synergy in combination therapy and long-term immune surveillance are needed to better characterize long-term benefits. While this study focused on chemically paired isotopes, future studies could evaluate the ability of [^64^Cu]-FAP to predict response to TRT, such as ^177^Lu, that are currently in clinical trials, as it provides longer half-life imaging compared to current [^68^Ga] and [^18^F] tracers used.

In conclusion, our study highlights the significance of TRT to modulate and sensitize the TME to increase treatment efficacy of IMT response and improve overall treatment outcomes. Using chemically paired radionuclides could help personalize treatment strategies and give a non-invasive approach to provide clinically relevant metrics for treatment selection in patients with TNBC patients, as Cu-64 imaging provide predictive capabilities of long-term survival from Cu-67 treatment. [^64/67^Cu]-FAP2287 TRT has potential to expand to several other cancer types, including pancreatic ductal adenocarcinoma, head and neck cancer, and colorectal cancer, which have high FAP expression in their TME.

## Supplementary Material

This is a list of supplementary files associated with this preprint. Click to download.

- CONFLICTOFINTEREST.docx
- Cu67TRTandIMTinTNBCsupplementalfiguresSS.pptx

## Figures and Tables

**Figure 1:**
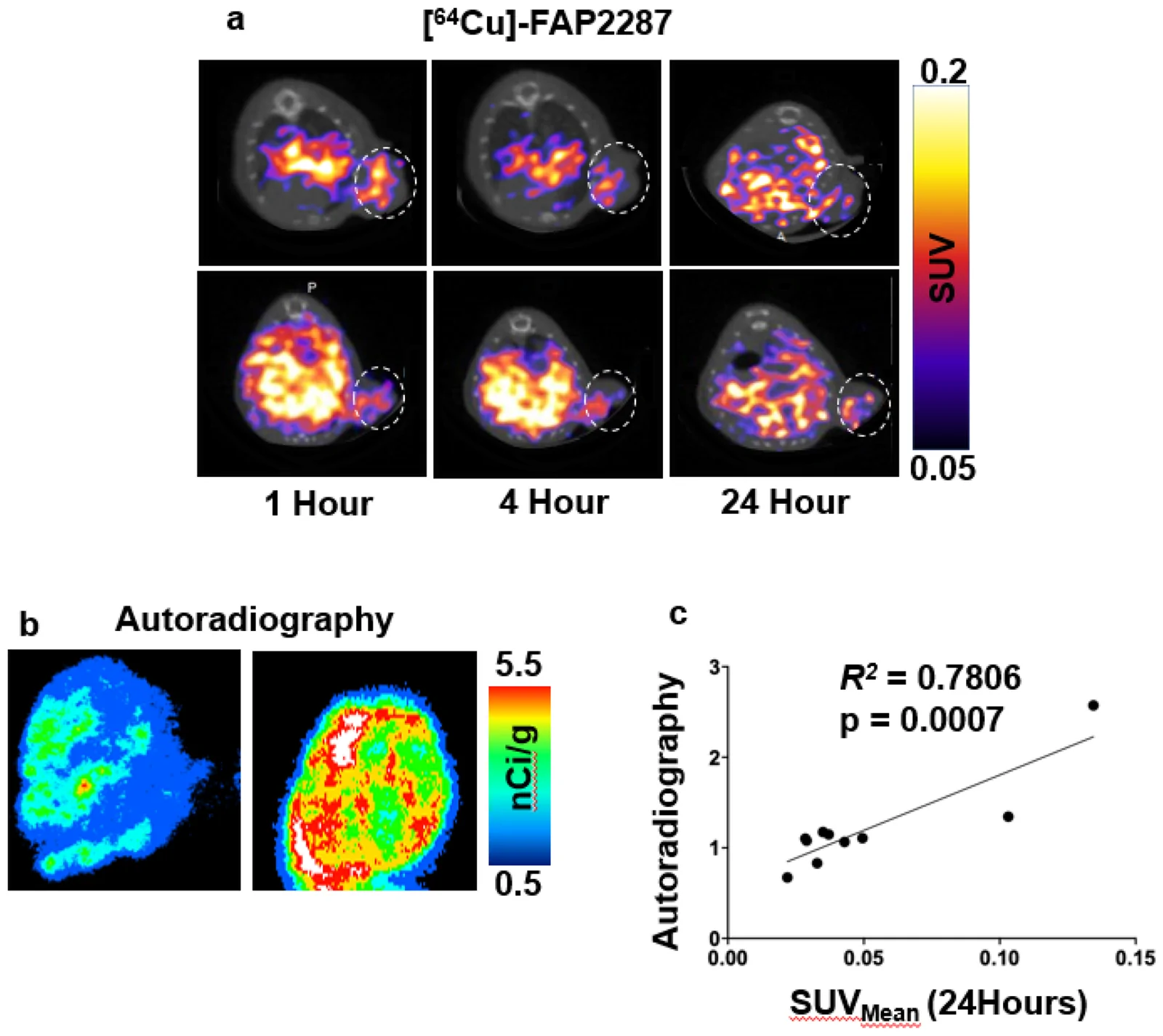
*Ex Vivo* biological validation of FAP expression in 4T1 TNBC model. (a) Representative transverse in vivo [^64^Cu]-FAP2287 PET imaging in 4T1 tumor bearing Balb/c mice at 4, 24 and 48h p.i. with the white dotted circle highlighting the tumor region. (b) Autoradiography showing radiotracer concentration the tumor region obtained following 24h imaging time point. (c) Strong Positive correlation between [^64^Cu]-FAP2287 SUV_Mean_ (24h) with radiotracer concentration recorded in autoradiography (R^2^=0.7806, p=0.0007)

**Figure 2:**
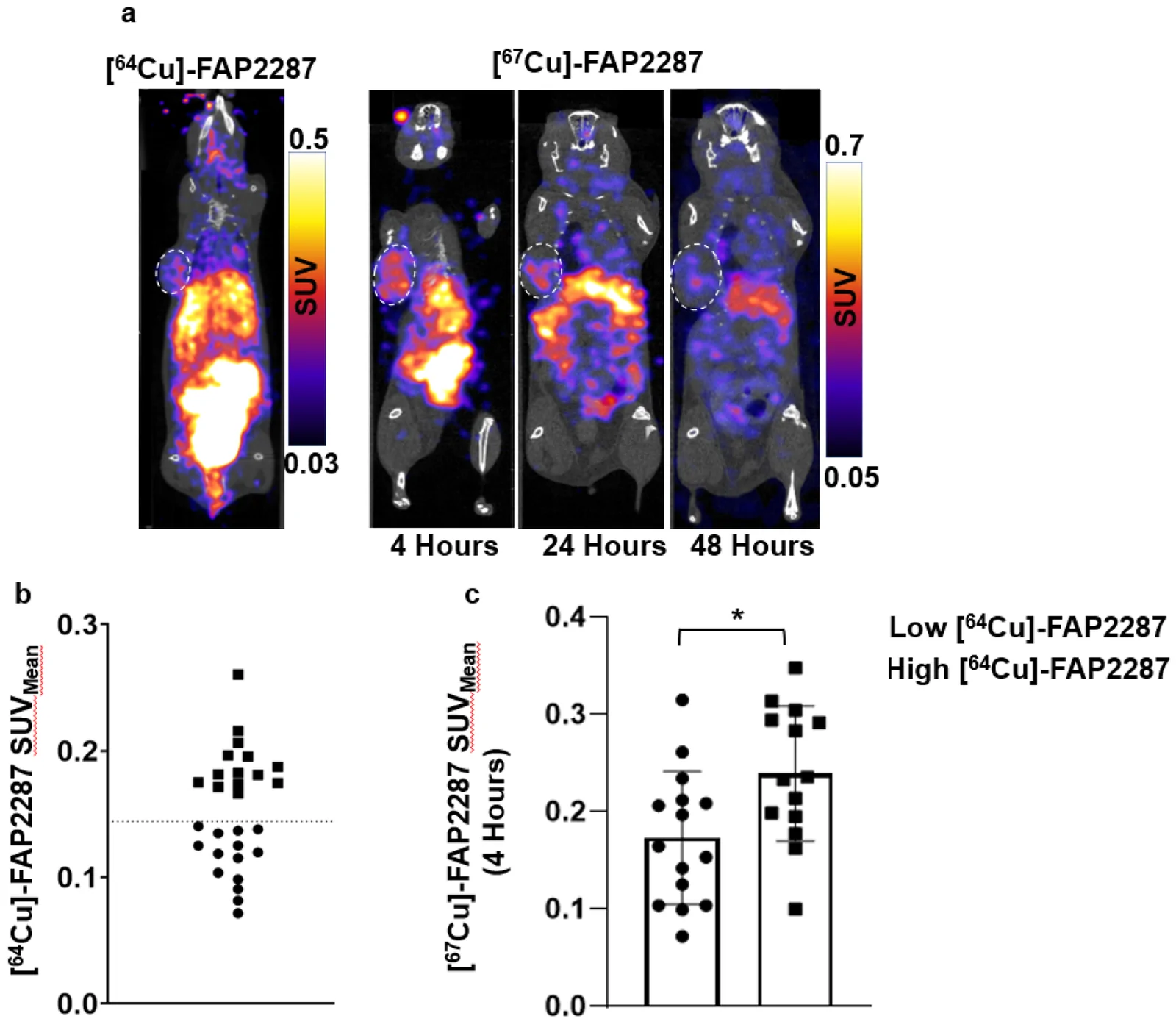
Positive correlation between chemically-paired radioisotopes [^64/67^Cu]-FAP2287. (a) Representative coronal images of [^64^Cu]-FAP2287 PET 4h p.i. and [^67^Cu]-FAP2287 SPECT 4, 24 and 48h p.i. showing quantifiable radiotracer uptake and increased retention in the tumor region highlighted by the white dotted circle. (b) Graphical representation of thresholding of mice into higher and lower [^64^Cu]-FAP2287 SUV_Mean_ (c) Graphical representation showing significant differences in [^67^Cu]-FAP2287 SUV_Mean_ (4h p.i.) between groups of low and high [^64^Cu]-FAP2287 SUV_Mean_

**Figure 3:**
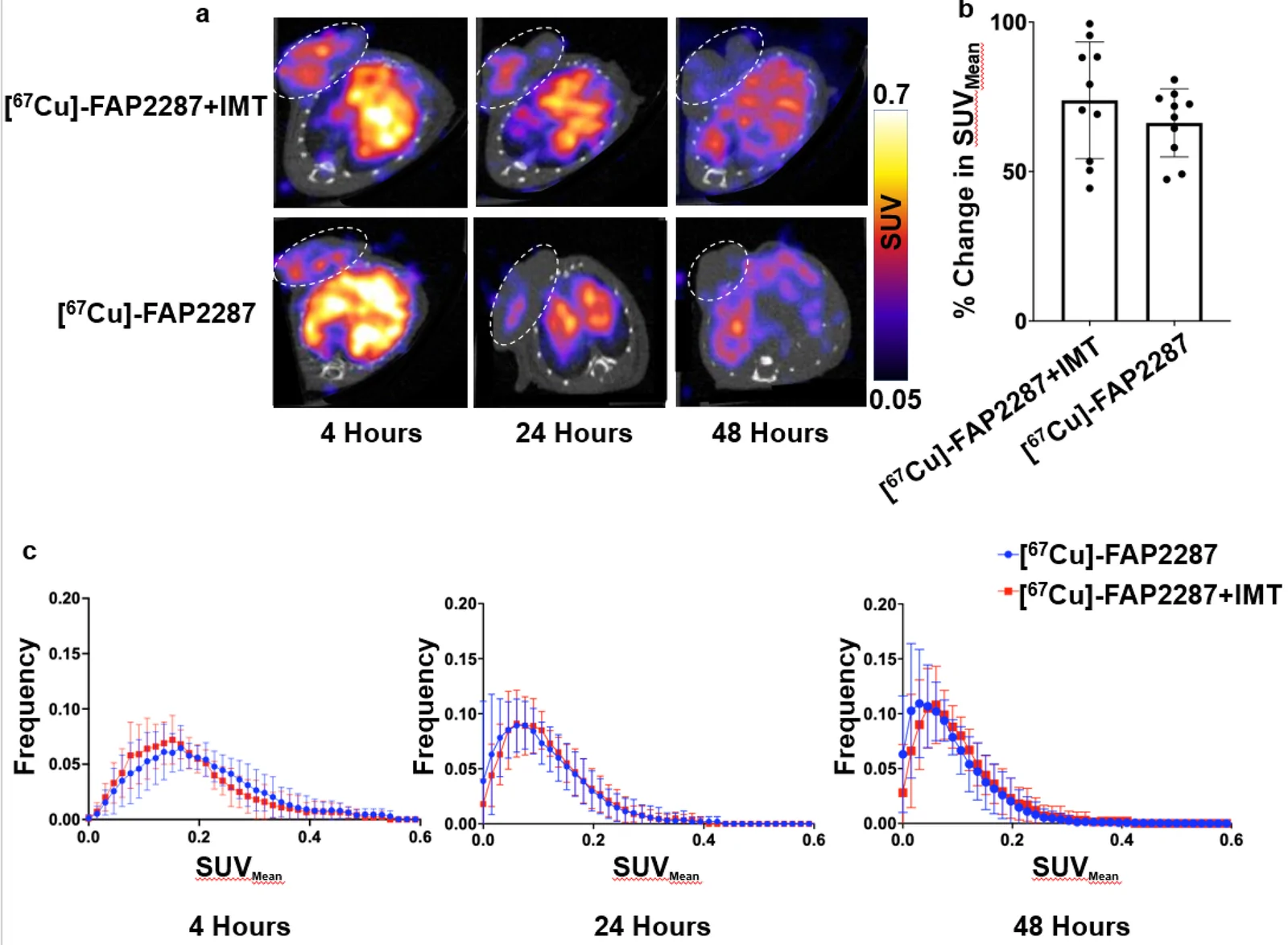
Effects of IMT in [^67^Cu]-FAP2287 retention. (a) Transverse [^67^Cu]-FAP2287 SPECT images at 4, 24 and 48h p.i., there was no difference in [^67^Cu]-FAP2287 retention between the groups in the tumor region highlighted by the white dotted circle. (b) Graphical representation of % retention of radiotracer 48h p.i. showing no statistical differences with the addition of IMT. (c) Histogram analysis of SUV_Mean_ indicating similar frequency distribution in both the groups

**Figure 4:**
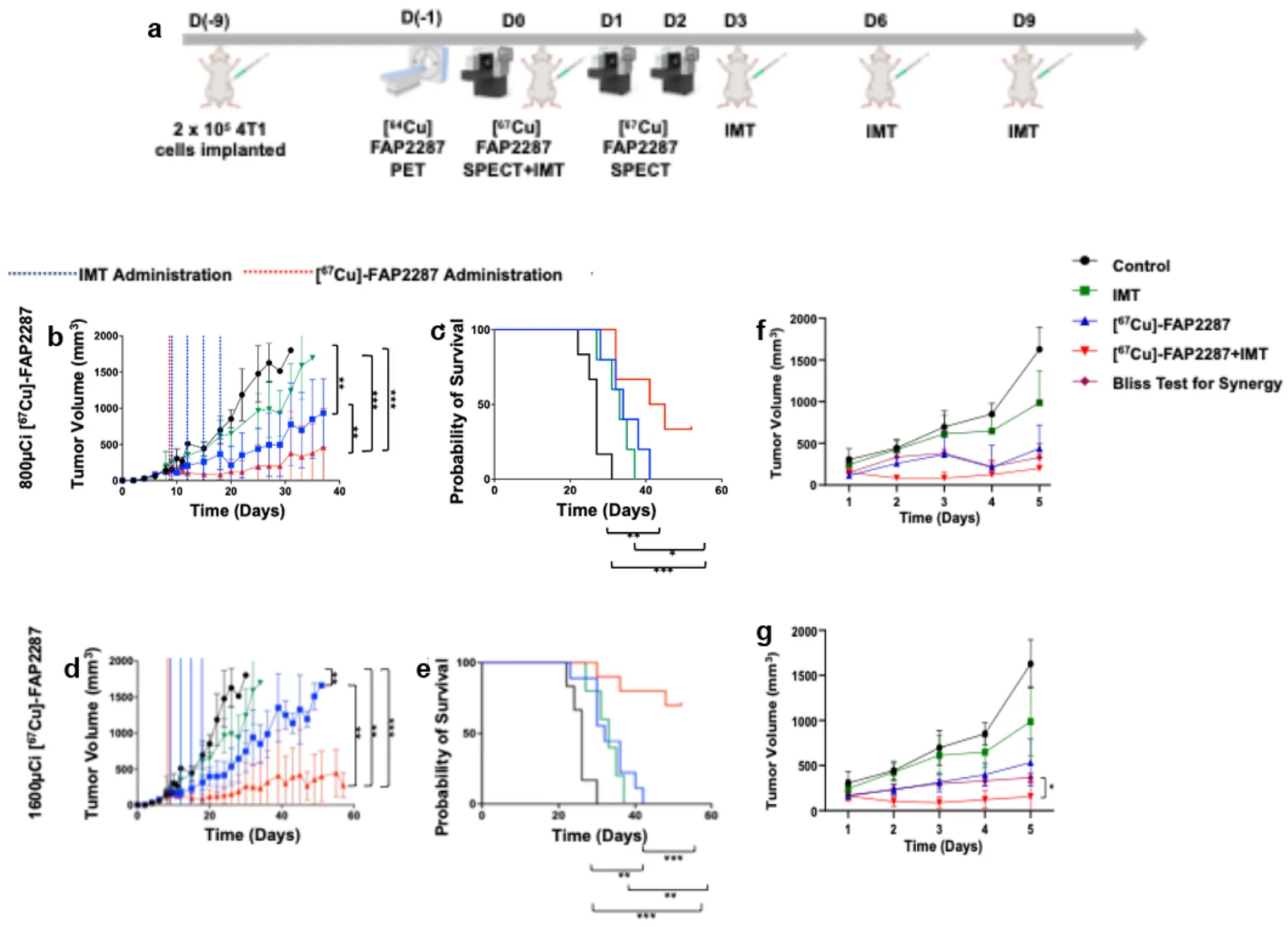
Response of 4T1 tumors to [^67^Cu]-FAP2287 in combination with IMT. (a) Experimental timeline for treatment. Tumor growth curve in 4T1 model administered with (b) 800μCi (d) 1600μCi [^67^Cu]-FAP2287 suggesting that higher dose of [^67^Cu]-FAP2287 significant decreases tumor burden in the combination group compared to single treatment groups. Probability of survival with (c) 800μCi (e) 1600μCi [^67^Cu]-FAP2287 indicating significant increase in probability of survival with 1600μCi combination group compared to all the other groups. (f) 800μCi (g) 1600μCi observed tumor volume in all treatment groups at five different time points and expected tumor volumes of the combination groups from the bliss test of synergy showing higher tumor volume compared to the observed combination tumor volumes suggesting synergy in the combination treatment

**Figure 5:**
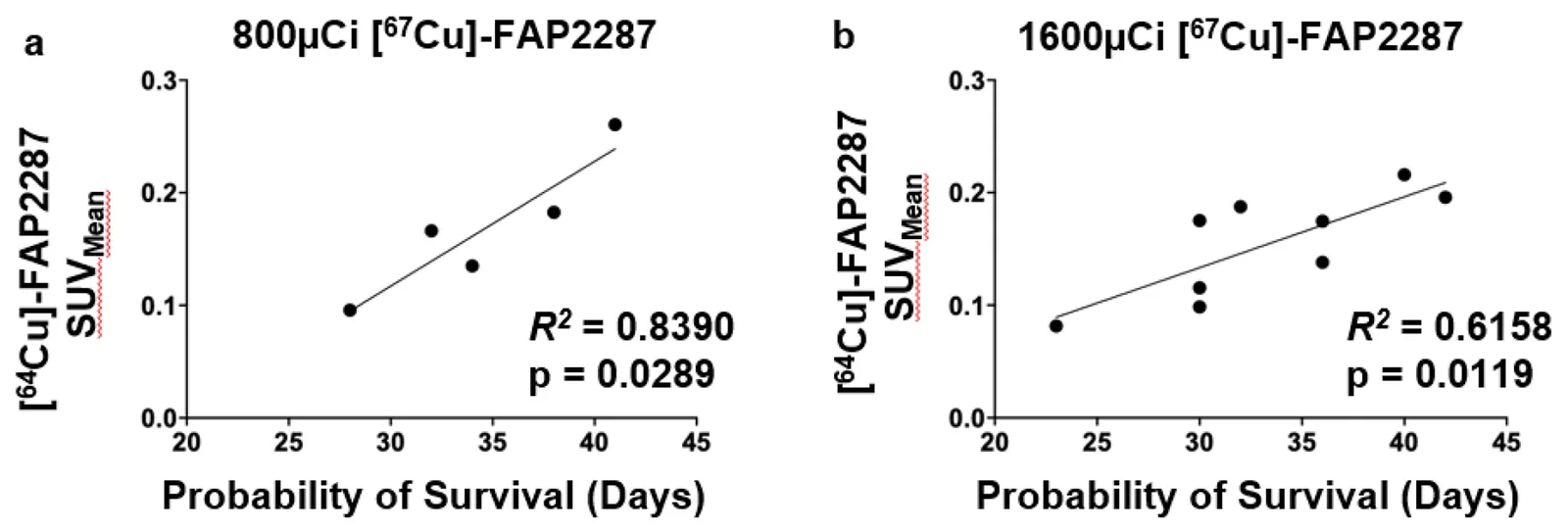
[^64^Cu]-FAP2287 PET imaging as a reliable predictor for response to [^67^Cu]-FAP2287 TRT. Positive correlation trend between probability of survival and [^64^Cu]-FAP2287 SUV_Mean_ in mice treated with (a) 800uCi of [^67^Cu]-FAP2287 (R^2^=0.839, p=0.0289) and (b) 1600uCi of [^67^Cu]-FAP2287 (R^2^=0.6158, p=0.0119) showing significant strong positive correlation between SUV_Mean_ and median survival in both cohorts

## Data Availability

Data generated in this study will be made available upon request.

## Abbreviations

TNBC: triple-negative breast cancer
FAP: fibroblast activation protein
TRT: Targeted Radionuclide Therapy
PET: Positron Emission Tomography
SPECT: Single Photon Emission Computed Tomography
TME: Tumor Microenvironment
IMT: Immunotherapy
CTLA-4: cytotoxic T-lymphocyte associated antigen 4
PD-1: programmed cell death receptor 1
ICD: Immunogenic cell death
CAF: Cancer associated fibroblast
SUV: Standardize uptake value VQ:Vivo Quant
TMR: tumor to muscle ratio
ROI: Regions of interest
CT: Computed tomography
BLI: Bioluminescence Imaging

## References

[1] Sia J, Szmyd R, Hau E, Gee HE (2020) Molecular Mechanisms of Radiation-Induced Cancer Cell Death: A Primer. Front Cell Dev Biol. ;8 10.3389/fcell.2020.00041 PMC7031160 32117972

[2] Obidiro O, Battogtokh G, Akala EO (2023) Triple Negative Breast Cancer Treatment Options and Limitations: Future Outlook. Pharmaceutics. ;15(7) 10.3390/pharmaceutics15071796 PMC10384267 37513983

[3] Dass SA, Tan KL, Rajan RS, Mokhtar NF, Adzmi ERM, Rahman WFWA (2021) Triple Negative Breast Cancer: A Review of Present and Future Diagnostic Modalities. Medicina-Lithuania. ;57(1) 10.3390/medicina57010062 PMC7826673 33445543

[4] Aysola K, Desai A, Welch C, Xu J, Qin Y, Reddy V (2013) Triple Negative Breast Cancer - An Overview. Hereditary Genet. ;2013(Suppl 2). 10.4172/2161-1041.S2-001 PMC4181680 25285241

[5] Ensenyat-Mendez M, Llinàs-Arias P, Orozco JIJ, Íñiguez-Muñoz S, Salomon MP, Sesé B (2021) Current Triple-Negative Breast Cancer Subtypes: Dissecting the Most Aggressive Form of Breast Cancer. Front Oncol 11:681476 10.3389/fonc.2021.681476 PMC8242253 34221999

[6] Zagami P, Carey LA (2022) Triple negative breast cancer: Pitfalls and progress. NPJ Breast Cancer 8(1):95 10.1038/s41523-022-00468-0 PMC9392735 35987766

[7] Cleator S, Heller W, Coombes RC (2007) Triple-negative breast cancer: therapeutic options. Lancet Oncol 8(3):235–244 10.1016/S1470-2045(07)70074-8 17329194

[8] Zhang Y, Zhang Z (2020) The history and advances in cancer immunotherapy: understanding the characteristics of tumor-infiltrating immune cells and their therapeutic implications. Cell Mol Immunol 17(8):807–821 10.1038/s41423-020-0488-6 PMC7395159 32612154

[9] Peterson C, Denlinger N, Yang Y (2022) Recent Advances and Challenges in Cancer Immunotherapy. Cancers (Basel). ;14(16) 10.3390/cancers14163972 PMC9406446 36010965

[10] Nadal E, Massuti B, Dómine M, García-Campelo R, Cobo M, Felip E (2019) Immunotherapy with checkpoint inhibitors in non-small cell lung cancer: insights from long-term survivors. Cancer Immunol Immunother 68(3):341–352 10.1007/s00262-019-02310-2 PMC11028247 30725206

[11] Hodi FS, O’Day SJ, McDermott DF, Weber RW, Sosman JA, Haanen JB (2010) Improved survival with ipilimumab in patients with metastatic melanoma. N Engl J Med 363(8):711–723 10.1056/NEJMoa1003466 PMC3549297 20525992

[12] Schadendorf D, Hodi FS, Robert C, Weber JS, Margolin K, Hamid O (2015) Pooled Analysis of Long-Term Survival Data From Phase II and Phase III Trials of Ipilimumab in Unresectable or Metastatic Melanoma. J Clin Oncol 33(17):1889–1894 10.1200/JCO.2014.56.2736 PMC5089162 25667295

[13] Rotte A (2019) Combination of CTLA-4 and PD-1 blockers for treatment of cancer. J Exp Clin Cancer Res 38(1):255 10.1186/s13046-019-1259-z PMC6567914 31196207

[14] Kumar P, Aggarwal R (2016) An overview of triple-negative breast cancer. Arch Gynecol Obstet 293(2):247–269 10.1007/s00404-015-3859-y 26341644

[15] Dent R, Trudeau M, Pritchard KI, Hanna WM, Kahn HK, Sawka CA (2007) Triple-negative breast cancer: clinical features and patterns of recurrence. Clin Cancer Res 13(15 Pt 1):4429–4434 10.1158/1078-0432.CCR-06-3045 17671126

[16] El-Tanani M, Rabbani SA, Babiker R, Rangraze I, Kapre S, Palakurthi SS (2024) Unraveling the tumor microenvironment: Insights into cancer metastasis and therapeutic strategies. Cancer Lett 591:216894 10.1016/j.canlet.2024.216894 38626856

[17] Balkwill FR, Capasso M, Hagemann T (2012) The tumor microenvironment at a glance. J Cell Sci 125(Pt 23):5591–5596 10.1242/jcs.116392 23420197

[18] Lotfinejad P, Kazemi T, Safaei S, Amini M, Roshani Asl E, Baghbani E (2021) PD-L1 silencing inhibits triple-negative breast cancer development and upregulates T-cell-induced pro-inflammatory cytokines. Biomed Pharmacother 138:111436 10.1016/j.biopha.2021.111436 33667790

[19] Ping Q, Yan R, Cheng X, Wang W, Zhong Y, Hou Z (2021) Cancer-associated fibroblasts: overview, progress, challenges, and directions. Cancer Gene Ther 28(9):984–999 10.1038/s41417-021-00318-4 33712707

[20] Shiga K, Hara M, Nagasaki T, Sato T, Takahashi H, Takeyama H (2015) Cancer-Associated Fibroblasts: Their Characteristics and Their Roles in Tumor Growth. Cancers (Basel) 7(4):2443–2458 10.3390/cancers7040902 PMC4695902 26690480

[21] Fitzgerald AA, Weiner LM (2020) The role of fibroblast activation protein in health and malignancy. Cancer Metastasis Rev 39(3):783–803 10.1007/s10555-020-09909-3 PMC7487063 32601975

[22] Piper M, Mueller AC, Karam SD (2020) The interplay between cancer associated fibroblasts and immune cells in the context of radiation therapy. Mol Carcinog 59(7):754–765 10.1002/mc.23205 PMC8011858 32363633

[23] Privé BM, Boussihmad MA, Timmermans B, van Gemert WA, Peters SMB, Derks YHW (2023) Fibroblast activation protein-targeted radionuclide therapy: background, opportunities, and challenges of first (pre)clinical studies. Eur J Nucl Med Mol Imaging 50(7):1906–1918 10.1007/s00259-023-06144-0 PMC10199876 36813980

[24] Zheng H, Siddharth S, Parida S, Wu X, Sharma D (2021) Tumor Microenvironment: Key Players in Triple Negative Breast Cancer Immunomodulation. Cancers (Basel) 13:13 10.3390/cancers13133357 PMC8269090 34283088

[25] Errarte P, Guarch R, Pulido R, Blanco L, Nunes-Xavier CE, Beitia M (2016) The Expression of Fibroblast Activation Protein in Clear Cell Renal Cell Carcinomas Is Associated with Synchronous Lymph Node Metastases. PLoS ONE 11(12):e0169105 10.1371/journal.pone.0169105 PMC5199084 28033421

[26] Kleinendorst SC, Oosterwijk E, Bussink J, Westdorp H, Konijnenberg MW, Heskamp S (2022) Combining Targeted Radionuclide Therapy and Immune Checkpoint Inhibition for Cancer Treatment. Clin Cancer Res 28(17):3652–3657 10.1158/1078-0432.CCR-21-4332 PMC9433955 35471557

[27] Keinänen O, Fung K, Brennan JM, Zia N, Harris M, van Dam E (2020) Harnessing (64)Cu/(67)Cu for a theranostic approach to pretargeted radioimmunotherapy. Proc Natl Acad Sci U S A 117(45):28316–28327 10.1073/pnas.2009960117 PMC7668034 33106429

[28] Zi F, He J, He D, Li Y, Yang L, Cai Z (2015) Fibroblast activation protein α in tumor microenvironment: recent progression and implications (review). Mol Med Rep 11(5):3203–3211 10.3892/mmr.2015.3197 PMC4368076 25593080

[29] Krönke T, Kopka K, Mamat C (2025) Enhancing the radionuclide theranostic concept through the radiohybrid approach. RSC Med Chem 16(5):1856–1864 10.1039/d4md00591k PMC11606402 39618962

[30] Danieli R, Milano A, Gallo S, Veronese I, Lascialfari A, Indovina L (2022) Personalized Dosimetry in Targeted Radiation Therapy: A Look to Methods, Tools and Critical Aspects. J Pers Med. ;12(2) 10.3390/jpm12020205 PMC8874397 35207693

[31] Miller C, Rousseau J, Ramogida CF, Celler A, Rahmim A, Uribe CF (2022) Implications of physics, chemistry and biology for dosimetry calculations using theranostic pairs. Theranostics 12(1):232–259 10.7150/thno.62851 PMC8690938 34987643

[32] Jansen K, Heirbaut L, Cheng JD, Joossens J, Ryabtsova O, Cos P (2013) Selective Inhibitors of Fibroblast Activation Protein (FAP) with a (4-Quinolinoyl)-glycyl-2-cyanopyrrolidine Scaffold. ACS Med Chem Lett 4(5):491–496 10.1021/ml300410d PMC4027141 24900696

[33] Kelkar SS, Reineke TM (2011) Theranostics: combining imaging and therapy. Bioconjug Chem 22(10):1879–1903 10.1021/bc200151q 21830812

[34] Zboralski D, Hoehne A, Bredenbeck A, Schumann A, Nguyen M, Schneider E (2022) Preclinical evaluation of FAP-2286 for fibroblast activation protein targeted radionuclide imaging and therapy. Eur J Nucl Med Mol Imaging 49(11):3651–3667 10.1007/s00259-022-05842-5 PMC9399058 35608703

[35] Zboralski D, Osterkamp F, Christensen E, Bredenbeck A, Schumann A, Hoehne A (2023) Fibroblast activation protein targeted radiotherapy induces an immunogenic tumor microenvironment and enhances the efficacy of PD-1 immune checkpoint inhibition. Eur J Nucl Med Mol Imaging 50(9):2621–2635 10.1007/s00259-023-06211-6 PMC10317891 37086273

[36] McCarthy DW, Shefer RE, Klinkowstein RE, Bass LA, Margeneau WH, Cutler CS (1997) Efficient production of high specific activity 64Cu using a biomedical cyclotron. Nucl Med Biol 24(1):35–43 10.1016/s0969-8051(96)00157-6 9080473

[37] Song PN, Mansur A, Dugger KJ, Davis TR, Howard G, Yankeelov TE (2020) CD4 T-cell immune stimulation of HER2 + breast cancer cells alters response to trastuzumab in vitro. Cancer Cell Int 20(1):544 10.1186/s12935-020-01625-w PMC7654187 33292267

[38] Gallegos CA, Lu Y, Clements JC, Song PN, Lynch SE, Mascioni A (2024) [(89)Zr]-CD8 ImmunoPET imaging of glioblastoma multiforme response to combination oncolytic viral and checkpoint inhibitor immunotherapy reveals CD8 infiltration differential changes in preclinical models. Theranostics 14(3):911–923 10.7150/thno.89206 PMC10797292 38250045

[39] Song PN, Lynch SE, DeMellier CT, Mansur A, Gallegos CA, Wright BD (2024) Dual anti-HER2/EGFR inhibition synergistically increases therapeutic effects and alters tumor oxygenation in HNSCC. Sci Rep 14(1):3771 10.1038/s41598-024-52897-5 PMC10866896 38355949

[40] Demidenko E, Miller TW (2019) Statistical determination of synergy based on Bliss definition of drugs independence. PLoS ONE 14(11):e0224137 10.1371/journal.pone.0224137 PMC6876842 31765385

[41] Ramon-Patino JL, Schmid S, Lau S, Seymour L, Gaudreau PO, Li JJN (2022) iRECIST and atypical patterns of response to immuno-oncology drugs. J Immunother Cancer. ;10(6) 10.1136/jitc-2022-004849 PMC9207898 35715004

[42] Yang1 L, Zhou1 T-y, Yong1 L, Zhu X 2 (2020) Feng1 Y, Fu1 Y,. Disease progression model of 4T1 metastatic breast cancer. Journal of Pharmacokinetics and Pharmacodynamics. ;47 10.1007/s10928-020-09673-5 31970615

[43] Chakravarty R, Song W, Chakraborty S, Cai W (2023) Fibroblast activation protein (FAP)-targeted radionuclide therapy: which ligand is the best? Eur J Nucl Med Mol Imaging 50(10):2935–2939 10.1007/s00259-023-06338-6 PMC10428190 37452872

[44] Hagemann UB, Ellingsen C, Schuhmacher J, Kristian A, Mobergslien A, Cruciani V (2019) Mesothelin-Targeted Thorium-227 Conjugate (MSLN-TTC): Preclinical Evaluation of a New Targeted Alpha Therapy for Mesothelin-Positive Cancers. Clin Cancer Res 25(15):4723–4734 10.1158/1078-0432.CCR-18-3476 31064781

[45] Gibbens-Bandala B, Morales-Avila E, Ferro-Flores G, Santos-Cuevas C, Meléndez-Alafort L, Trujillo-Nolasco M (2019) (177)Lu-Bombesin-PLGA (paclitaxel): A targeted controlled-release nanomedicine for bimodal therapy of breast cancer. Mater Sci Eng C Mater Biol Appl 105:110043 10.1016/j.msec.2019.110043 31546458

[46] Kasten BB, Oliver PG, Kim H, Fan J, Ferrone S, Zinn KR (2018) (212)Pb-Labeled Antibody 225.28 Targeted to Chondroitin Sulfate Proteoglycan 4 for Triple-Negative Breast Cancer Therapy in Mouse Models. Int J Mol Sci. ;19(4) 10.3390/ijms19040925 PMC5979285 29561763

[47] Kerr CP, Grudzinski JJ, Nguyen TP, Hernandez R, Weichert JP, Morris ZS (2022) Developments in Combining Targeted Radionuclide Therapies and Immunotherapies for Cancer Treatment. Pharmaceutics. ;15(1) 10.3390/pharmaceutics15010128 PMC9865370 36678756

[48] Vito A, Rathmann S, Mercanti N, El-Sayes N, Mossman K, Valliant J (2021) Combined Radionuclide Therapy and Immunotherapy for Treatment of Triple Negative Breast Cancer. Int J Mol Sci. ;22(9) 10.3390/ijms22094843 PMC8124136 34063642

[49] Roya Eisazadeh SAM, Gregor Schwieghofer-Zwink L, Hehenwarter G, Rendl S, Gampenrieder R, Greil C, Pirich (2024) Mohsen Beheshti. Pre-treatment 68 Ga-PSMA-11 PET/CT Prognostic Value in Predicting Response to 177Lu-PSMA-I&T Therapy and Patient Survival. Mol Imaging Biology 26(2):10 10.1007/s11307-024-01900-6 PMC10973080 38360991

[50] Ozkan a BDb E, Soydal a C, Araz a M, Dursun a E, Kucuk a NO (2025) Y. Urun c. Prediction of [177Lu]Lu-PSMA-617 treatment response with [68Ga]Ga-PSMA-11 PET-derived variables and correlation of the overall survival with serum PSA and PSMA PET-based response criteriaPredicción de la respuesta al tratamiento con [177Lu]Lu-PSMA-617 mediante variables derivadas de [68Ga]Ga-PSMA-11 PET y correlación de la supervivencia global con el PSA sérico y los criterios de respuesta basados en PSMA PET. Rev Esp Med Nucl Imagen Mol (Engl Ed) 10.1016/j.remnie.2025.500208 40998007

[51] Al-Nahhasb ∙ Eric O (2019) Aboagyea ∙ Tara D. Barwicka,b RSrsiauWMWSYJERRFWAFFMA. 68Ga-DOTATATE PET/CT parameters predict response to peptide receptor radionuclide therapy in neuroendocrine tumours. Radiother Oncol 141:8 10.1016/j.radonc.2019.09.003 31542317

[52] Hwan Lee1 STK, Remy Niman, Sophia R, O’Brien JR, Eads BW (2023) Katona, and Daniel A. Pryma. Prediction of 177Lu-DOTATATE Therapy Outcomes in Neuroendocrine Tumor Patients Using Semi-Automatic Tumor Delineation on 68Ga-DOTATATE PET/CT. Cancers (Basel). ;16(1) 10.3390/cancers16010200 PMC10778298 38201627

[53] Lapi SJCPAHRGSTE (2026) Investigating the Theranostic Potential of Elementally Matched [43Sc]Sc-PSMA-617 and [47Sc]Sc-PSMA-617. Mol pharmaceuticals. ;23(3) 10.1021/acs.molpharmaceut.5c01023 PMC12958282 41705479

